# (*E*)-4-Bromo-*N*-(2,4-dimethoxy­benzyl­idene)aniline

**DOI:** 10.1107/S1600536809004905

**Published:** 2009-02-21

**Authors:** Aliakbar D. Khalaji, Jim Simpson

**Affiliations:** a Department of Chemistry, Faculty of Science, Golestan University, Gorgan, Iran; b Department of Chemistry, University of Otago, PO Box 56, Dunedin, New Zealand

## Abstract

The title Schiff base compound, C_15_H_14_BrNO_2_, adopts an *E* configuration with respect to the C=N bond. The C and O atoms of the two meth­oxy substituents lie very close to the dimethoxy­phenyl ring plane [maximum deviation = 0.17 (1) Å]. The dihedral angle between the two aromatic rings is 43.69 (16)°, while the plane through the central C—C=N—C system is inclined at 10.6 (6)° to the dimethoxy­phenyl ring and 34.6 (3)° to the bromo­phenyl ring. In the crystal structure, each mol­ecule is involved in the formation of two inversion-related dimers through weak C—H⋯N and C—H⋯O inter­actions, respectively. These contacts link the mol­ecules into independent rows parallel to the *b* axis.

## Related literature

For related structures, see: Khalaji *et al.* (2007 ▶); Khalaji & Harrison (2008 ▶); Khalaji & Simpson (2009 ▶). For reference structural data, see: Allen *et al.* (1987 ▶). For graph-set motifs, see: Bernstein *et al.* (1995 ▶).
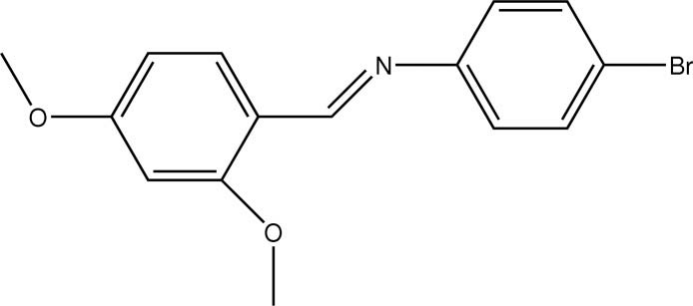


## Experimental

### 

#### Crystal data

C_15_H_14_BrNO_2_
*M* *_r_* = 320.18
Monoclinic, 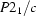

*a* = 4.1323 (6) Å
*b* = 10.7406 (14) Å
*c* = 29.911 (4) Å
β = 90.992 (8)°
*V* = 1327.4 (3) Å^3^
*Z* = 4
Mo *K*α radiation
μ = 3.09 mm^−1^
*T* = 89 K
0.25 × 0.10 × 0.02 mm

#### Data collection

Bruker APEXII CCD area-detector diffractometer
Absorption correction: multi-scan (*SADABS*; Bruker, 2006 ▶) *T*_min_ = 0.570, *T*_max_ = 0.940
13728 measured reflections
2390 independent reflections
1664 reflections with *I* > 2σ(*I*)
*R*_int_ = 0.106

#### Refinement

*R*[*F* ^2^ > 2σ(*F* ^2^)] = 0.056
*wR*(*F* ^2^) = 0.118
*S* = 1.21
2390 reflections
174 parameters
H-atom parameters constrained
Δρ_max_ = 0.83 e Å^−3^
Δρ_min_ = −0.82 e Å^−3^

### 

Data collection: *APEX2* (Bruker, 2006 ▶); cell refinement: *APEX2* and *SAINT* (Bruker, 2006 ▶); data reduction: *SAINT*; program(s) used to solve structure: *SHELXS97* (Sheldrick, 2008 ▶); program(s) used to refine structure: *SHELXL97* (Sheldrick, 2008 ▶) and *TITAN2000* (Hunter & Simpson, 1999 ▶); molecular graphics: *SHELXTL* (Sheldrick, 2008 ▶) and *Mercury* (Macrae *et al.*, 2006 ▶); software used to prepare material for publication: *SHELXL97*, *enCIFer* (Allen *et al.*, 2004 ▶), *PLATON* (Spek, 2009 ▶) and *publCIF* (Westrip, 2009 ▶).

## Supplementary Material

Crystal structure: contains datablocks global, I. DOI: 10.1107/S1600536809004905/ng2544sup1.cif

Structure factors: contains datablocks I. DOI: 10.1107/S1600536809004905/ng2544Isup2.hkl

Additional supplementary materials:  crystallographic information; 3D view; checkCIF report

## Figures and Tables

**Table 1:** Hydrogen-bond geometry (Å, °)

*D*—H⋯*A*	*D*—H	H⋯*A*	*D*⋯*A*	*D*—H⋯*A*
C7—H7*A*⋯N1^i^	0.98	2.74	3.667 (7)	159
C4—H4*C*⋯O2^ii^	0.98	2.54	3.398 (6)	145

Symmetry codes: (i) 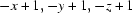; (ii) 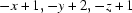.

**Fig. 1.**
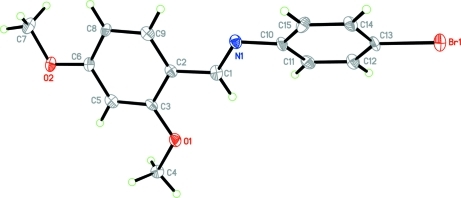
The structure of (I) with displacement ellipsoids for the non-hydrogen atoms drawn at the 50% probability level.

**Fig. 2.**
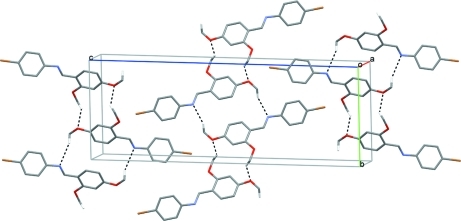
Crystal packing of (I) viewed down the a axis with hydrogen bonds drawn as dashed lines. H atoms not involved in hydrogen bonding have been omitted.

## supplementary crystallographic information

### Comment

As a continuation of our work on the synthesis and structural characterization of Schiff-base compounds (Khalaji *et al.*, 2007; Khalaji & Harrison, 2008; Khalaji & Simpson, 2009), we report here the structure of the title compound, C_15_H_14_BrNO_2_, (I), Fig 1.

The compound adopts an *E* configuration with respect to the C1=N1 bond. The C4, O1 and C7 O2 methoxy substituents lie close to the plane of the C2···C9 ring (maximum deviation 0.17 (1) Å for C7. Bond lengths in the molecule are normal (Allen, *et al.*, 1987) and similar to those found in related compounds (Khalaji *et al.*, 2007; Khalaji & Harrison, 2008; Khalaji & Simpson 2009). The dihedral angle between the two aromatic rings is 43.69 (16) ° while the plane through the central C2—C2?N1—C10 system is inclined at 10.6 (6)° to the dimethoxyphenyl ring and 34.6 (3)° to the bromobenzene ring.

In the crystal structure, each molecule is involved in the formation of two inversion related dimers with *R*^2^_2_(18) and *R*^2^_2_(14) ring motifs (Bernstein *et al.* 1995) through weak C7—H7A···N1 and C4—H4···O2 interactions respectively, Table 1. These contacts link the molecules into independent rows parallel to the *b* axis, Fig. 2.

### Experimental

To a solution of 2,4-Dimethoxy benzaldehyde (332 mg, 0.2 mmol) in methanol (5 ml), cooled in an ice bath, a solution of 4-bromoaniline (344 mg, 0.2 mmol) in methanol (5 ml) was added slowly dropwise with constant stirring (1 h) at 298 k in the presence of molecular sieves. The mixture was filtered and the solution cooled to 273 K to give the compound in about 85% yield. Pale yellow crystals were grown from methanol.

### Refinement

H-atoms were refined using a riding model with d(C—H) = 0.95 Å, *U*_iso_= 1.2*U*_eq_ (C) for aromatic and 0.98 Å, *U*_iso_ = 1.5*U*_eq_ (C) for CH_3_ H atoms.

### Crystal data

**Table:**

C_15_H_14_BrNO_2_	*F*(000) = 648
*M**_r_* = 320.18	*D*_x_ = 1.602 Mg m^−^^3^
Monoclinic, *P*2_1_/*c*	Mo *K*α radiation, λ = 0.71073 Å
Hall symbol: -P 2ybc	Cell parameters from 2351 reflections
*a* = 4.1323 (6) Å	θ = 2.7–23.6°
*b* = 10.7406 (14) Å	µ = 3.09 mm^−^^1^
*c* = 29.911 (4) Å	*T* = 89 K
β = 90.992 (8)°	Rectangular plate, pale yellow
*V* = 1327.4 (3) Å^3^	0.25 × 0.10 × 0.02 mm
*Z* = 4	

### Data collection

**Table:**

Bruker APEXII CCD area-detector diffractometer	2390 independent reflections
Radiation source: fine-focus sealed tube	1664 reflections with *I* > 2σ(*I*)
graphite	*R*_int_ = 0.106
ω scans	θ_max_ = 25.3°, θ_min_ = 2.0°
Absorption correction: multi-scan (*SADABS*; Bruker, 2006)	*h* = −3→4
*T*_min_ = 0.570, *T*_max_ = 0.940	*k* = −12→12
13728 measured reflections	*l* = −35→35

### Refinement

**Table:**

Refinement on *F*^2^	Primary atom site location: structure-invariant direct methods
Least-squares matrix: full	Secondary atom site location: difference Fourier map
*R*[*F*^2^ > 2σ(*F*^2^)] = 0.056	Hydrogen site location: inferred from neighbouring sites
*wR*(*F*^2^) = 0.118	H-atom parameters constrained
*S* = 1.21	*w* = 1/[σ^2^(*F*_o_^2^) + (0.0212*P*)^2^ + 3*P*] where *P* = (*F*_o_^2^ + 2*F*_c_^2^)/3
2390 reflections	(Δ/σ)_max_ < 0.001
174 parameters	Δρ_max_ = 0.83 e Å^−^^3^
0 restraints	Δρ_min_ = −0.82 e Å^−^^3^

### Special details

**Table:**

Geometry. All e.s.d.'s (except the e.s.d. in the dihedral angle between two l.s. planes) are estimated using the full covariance matrix. The cell e.s.d.'s are taken into account individually in the estimation of e.s.d.'s in distances, angles and torsion angles; correlations between e.s.d.'s in cell parameters are only used when they are defined by crystal symmetry. An approximate (isotropic) treatment of cell e.s.d.'s is used for estimating e.s.d.'s involving l.s. planes.
Refinement. Refinement of *F*^2^ against ALL reflections. The weighted *R*-factor *wR* and goodness of fit *S* are based on *F*^2^, conventional *R*-factors *R* are based on *F*, with *F* set to zero for negative *F*^2^. The threshold expression of *F*^2^ > σ(*F*^2^) is used only for calculating *R*-factors(gt) *etc*. and is not relevant to the choice of reflections for refinement. *R*-factors based on *F*^2^ are statistically about twice as large as those based on *F*, and *R*- factors based on ALL data will be even larger.

### Fractional atomic coordinates and isotropic or equivalent isotropic displacement parameters (Å^2^)

**Table:**

	*x*	*y*	*z*	*U*_iso_*/*U*_eq_	
N1	0.8952 (10)	0.5770 (4)	0.37825 (15)	0.0157 (10)	
C1	0.7282 (12)	0.6745 (5)	0.38778 (18)	0.0154 (12)	
H1	0.6759	0.7321	0.3647	0.018*	
C2	0.6171 (12)	0.6989 (5)	0.43302 (17)	0.0138 (12)	
C3	0.4148 (12)	0.8016 (5)	0.44237 (18)	0.0133 (12)	
O1	0.3071 (9)	0.8689 (3)	0.40592 (12)	0.0174 (9)	
C4	0.1171 (13)	0.9771 (4)	0.41475 (18)	0.0159 (12)	
H4A	−0.0826	0.9525	0.4296	0.024*	
H4B	0.0632	1.0192	0.3865	0.024*	
H4C	0.2406	1.0339	0.4342	0.024*	
C5	0.3327 (12)	0.8293 (5)	0.48586 (18)	0.0155 (12)	
H5	0.2003	0.8994	0.4919	0.019*	
C6	0.4459 (12)	0.7535 (5)	0.52094 (17)	0.0138 (12)	
O2	0.3510 (9)	0.7888 (3)	0.56290 (11)	0.0167 (9)	
C7	0.4941 (14)	0.7232 (5)	0.59995 (18)	0.0222 (13)	
H7A	0.4270	0.6357	0.5989	0.033*	
H7B	0.4228	0.7607	0.6280	0.033*	
H7C	0.7304	0.7283	0.5984	0.033*	
C8	0.6428 (12)	0.6515 (5)	0.51264 (17)	0.0153 (12)	
H8	0.7184	0.5999	0.5364	0.018*	
C9	0.7263 (12)	0.6267 (5)	0.46874 (18)	0.0160 (12)	
H9	0.8634	0.5578	0.4629	0.019*	
C10	1.0177 (13)	0.5654 (6)	0.33449 (18)	0.0169 (13)	
C11	1.1201 (12)	0.6657 (5)	0.30882 (18)	0.0181 (13)	
H11	1.0997	0.7481	0.3200	0.022*	
C12	1.2516 (13)	0.6466 (5)	0.26700 (18)	0.0179 (12)	
H12	1.3267	0.7153	0.2501	0.022*	
C13	1.2726 (12)	0.5278 (5)	0.25015 (18)	0.0164 (13)	
Br1	1.44896 (13)	0.49899 (7)	0.192736 (17)	0.02466 (19)	
C14	1.1702 (12)	0.4254 (5)	0.27488 (19)	0.0172 (12)	
H14	1.1866	0.3432	0.2633	0.021*	
C15	1.0447 (13)	0.4469 (6)	0.31650 (18)	0.0176 (13)	
H15	0.9738	0.3779	0.3336	0.021*	

### Atomic displacement parameters (Å^2^)

**Table:**

	*U*^11^	*U*^22^	*U*^33^	*U*^12^	*U*^13^	*U*^23^
N1	0.018 (2)	0.013 (3)	0.017 (2)	0.000 (2)	0.004 (2)	−0.0016 (19)
C1	0.018 (3)	0.011 (3)	0.017 (3)	−0.005 (2)	0.002 (2)	−0.003 (2)
C2	0.014 (3)	0.009 (3)	0.018 (3)	−0.001 (2)	0.003 (2)	−0.002 (2)
C3	0.008 (3)	0.013 (3)	0.019 (3)	0.000 (2)	0.001 (2)	−0.003 (2)
O1	0.020 (2)	0.016 (2)	0.016 (2)	0.0038 (17)	0.0036 (16)	−0.0012 (16)
C4	0.022 (3)	0.006 (3)	0.021 (3)	0.001 (2)	0.002 (2)	0.002 (2)
C5	0.015 (3)	0.008 (3)	0.024 (3)	−0.003 (2)	0.003 (2)	−0.003 (2)
C6	0.016 (3)	0.012 (3)	0.014 (3)	−0.006 (2)	0.002 (2)	−0.002 (2)
O2	0.023 (2)	0.015 (2)	0.012 (2)	−0.0021 (17)	0.0038 (16)	−0.0013 (16)
C7	0.036 (3)	0.015 (3)	0.015 (3)	−0.001 (3)	0.004 (3)	0.000 (2)
C8	0.017 (3)	0.016 (3)	0.013 (3)	−0.002 (2)	0.003 (2)	0.006 (2)
C9	0.014 (3)	0.012 (3)	0.022 (3)	−0.003 (2)	0.001 (2)	−0.003 (2)
C10	0.017 (3)	0.020 (3)	0.014 (3)	0.003 (2)	0.003 (2)	−0.005 (2)
C11	0.018 (3)	0.012 (3)	0.024 (3)	0.002 (2)	0.003 (2)	−0.002 (2)
C12	0.018 (3)	0.014 (3)	0.022 (3)	−0.003 (2)	0.007 (2)	0.002 (2)
C13	0.014 (3)	0.020 (4)	0.016 (3)	0.002 (2)	0.006 (2)	−0.005 (2)
Br1	0.0271 (3)	0.0313 (3)	0.0159 (3)	0.0036 (3)	0.0069 (2)	−0.0002 (3)
C14	0.020 (3)	0.009 (3)	0.022 (3)	0.002 (2)	0.005 (2)	−0.002 (2)
C15	0.022 (3)	0.016 (3)	0.014 (3)	−0.005 (2)	0.000 (3)	0.003 (2)

### Geometric parameters (Å, °)

**Table:**

N1—C1	1.288 (7)	C7—H7A	0.9800
N1—C10	1.417 (7)	C7—H7B	0.9800
C1—C2	1.460 (7)	C7—H7C	0.9800
C1—H1	0.9500	C8—C9	1.389 (7)
C2—C9	1.389 (7)	C8—H8	0.9500
C2—C3	1.415 (7)	C9—H9	0.9500
C3—O1	1.376 (6)	C10—C15	1.387 (8)
C3—C5	1.383 (7)	C10—C11	1.393 (8)
O1—C4	1.430 (6)	C11—C12	1.388 (7)
C4—H4A	0.9800	C11—H11	0.9500
C4—H4B	0.9800	C12—C13	1.375 (7)
C4—H4C	0.9800	C12—H12	0.9500
C5—C6	1.402 (7)	C13—C14	1.396 (7)
C5—H5	0.9500	C13—Br1	1.902 (5)
C6—O2	1.375 (6)	C14—C15	1.376 (8)
C6—C8	1.390 (7)	C14—H14	0.9500
O2—C7	1.432 (6)	C15—H15	0.9500
			
C1—N1—C10	118.5 (5)	O2—C7—H7C	109.5
N1—C1—C2	122.0 (5)	H7A—C7—H7C	109.5
N1—C1—H1	119.0	H7B—C7—H7C	109.5
C2—C1—H1	119.0	C6—C8—C9	118.4 (5)
C9—C2—C3	117.9 (5)	C6—C8—H8	120.8
C9—C2—C1	120.6 (5)	C9—C8—H8	120.8
C3—C2—C1	121.3 (5)	C2—C9—C8	122.5 (5)
O1—C3—C5	123.4 (5)	C2—C9—H9	118.7
O1—C3—C2	115.9 (4)	C8—C9—H9	118.7
C5—C3—C2	120.6 (5)	C15—C10—C11	117.9 (5)
C3—O1—C4	116.9 (4)	C15—C10—N1	118.1 (5)
O1—C4—H4A	109.5	C11—C10—N1	123.9 (5)
O1—C4—H4B	109.5	C12—C11—C10	120.6 (5)
H4A—C4—H4B	109.5	C12—C11—H11	119.7
O1—C4—H4C	109.5	C10—C11—H11	119.7
H4A—C4—H4C	109.5	C13—C12—C11	119.7 (5)
H4B—C4—H4C	109.5	C13—C12—H12	120.1
C3—C5—C6	119.7 (5)	C11—C12—H12	120.1
C3—C5—H5	120.2	C12—C13—C14	121.0 (5)
C6—C5—H5	120.2	C12—C13—Br1	120.6 (4)
O2—C6—C8	123.9 (5)	C14—C13—Br1	118.3 (4)
O2—C6—C5	115.2 (5)	C15—C14—C13	118.0 (5)
C8—C6—C5	120.9 (5)	C15—C14—H14	121.0
C6—O2—C7	116.8 (4)	C13—C14—H14	121.0
O2—C7—H7A	109.5	C14—C15—C10	122.6 (5)
O2—C7—H7B	109.5	C14—C15—H15	118.7
H7A—C7—H7B	109.5	C10—C15—H15	118.7

### Hydrogen-bond geometry (Å, °)

**Table:**

*D*—H···*A*	*D*—H	H···*A*	*D*···*A*	*D*—H···*A*
C7—H7A···N1^i^	0.98	2.74	3.667 (7)	159
C4—H4C···O2^ii^	0.98	2.54	3.398 (6)	145

Symmetry codes: (i) −*x*+1, −*y*+1, −*z*+1; (ii) −*x*+1, −*y*+2, −*z*+1.
